# From information to existence: the Information–Meaning–Existence (IBE) framework as a meta-method for second-order observation

**DOI:** 10.3389/fsoc.2026.1871301

**Published:** 2026-09-04

**Authors:** Hans Mund

**Affiliations:** Independent Researcher, Bergheim, Germany

**Keywords:** active inference, AI-assisted text analysis, computational social science, *in-silico* sociology, Luhmann, second-order observation, systems theory, validation protocol

## Abstract

Sociological systems theory offers a rich vocabulary for meaning, observation, and communication, but that vocabulary has proven difficult to translate into executable, testable, and revisable procedures. This gap has become more consequential as large language models are increasingly used to read, summarize, and simulate social-scientific discourse without any shared standard for distinguishing genuine semantic stabilization from fluent but ungrounded continuation. This paper introduces the Information–Meaning–Existence (IBE) framework, which reconstructs Luhmannian second-order observation as a validation architecture. A coherence index tracks whether actualized meaning-relations remain viable as their context changes, and a companion diagnostic identifies when that viability collapses—distinguishing collapse as failure from collapse as a precondition for new distinctions. The framework specifies how its central quantities could be computed from text using existing embedding-based and information-theoretic methods, disambiguates several senses in which a meaning-structure can be said to “exist,” and extends the coherence index to register cases in which apparent stability is sustained by structural power rather than genuine resonance. A concluding discussion confronts this coherence-centered vocabulary with dialectical accounts of development in which contradiction, not coherence, drives change, and uses that confrontation to recalibrate the framework's own claims. The paper specifies what an empirical pilot would require to calibrate and test the framework, and explicitly states that absent such calibration, its present status is proto-scientific.

## 1 Introduction: from metaphor to validation engine

Systems theory has long supplied sociology with a vocabulary powerful enough to describe complexity, self-reference, and meaning. What it has supplied less often is a method rigorous enough to test them. One can speak of autopoiesis, of structural coupling, of observation observing observation. The words remain strong. Their operational status often remains vague.

That is the central problem this paper addresses. Sociological systems research has repeatedly called for a formal and information-theoretic operationalization of Luhmann's concept of meaning (Leydesdorff, 1996). Yet many attempts have remained at the level of descriptive metaphor, preserving the prestige of the concepts without translating them into parameters that can be made explicit, varied, stressed, and assessed. If meaning is central to social systems, then sociology requires more than admiration for semantic subtlety. It requires criteria under which structures of meaning can be said to remain viable, to become fragile, or to collapse into noise. This requirement has become more, not less, urgent with the proliferation of large language models that can generate fluent commentary on social phenomena without any explicit account of when that commentary stabilizes a distinction and when it merely simulates the appearance of doing so.

The thesis of this paper is simple, though not small: Luhmann's observational program reaches its greatest methodological force when reconstructed as an operationalizable test design. Building on Baecker's (1993) formalization of form as a sociological calculus of operations and grounding the informational side of the argument in Shannon (1948), the present paper proposes that meaning must be translated into testable effectiveness without being reduced to flat state description.

The solution developed here is the IBE framework: Information—Meaning—Existence. IBE is not offered as a total theory of society. It is narrower, and therefore more demanding. It functions as an instrumental theory and a validation architecture. Its task is to evaluate whether distinctions, once actualized in discourse, generate contextual resonance and observable effects—or whether they merely simulate coherence while amplifying semantic drift.

The wager of the paper is deliberately restrained. Meaning is not treated as a substance hidden behind discourse, waiting to be extracted. Nor is it collapsed into a naive measurable quantity. What can be measured is not the fullness of possible meaning. What can be measured is the local viability of actualized relations within an informational field. The rest remains contingency. It remains there on purpose.

### 1.1 Scope of the present paper

A framework that simultaneously formalizes systems theory, operationalizes meaning, proposes a computational protocol, criticizes the governance of AI-assisted research, introduces a methodology, and offers an illustrative application risks doing all of these things badly. The present paper therefore restricts itself deliberately. Three commitments follow from this restriction.

First, the paper's mission can be stated in a single sentence: *to specify the formal architecture, the operational metrics, and the falsification criteria under which Luhmannian meaning-stabilization can be tested—by humans or by AI-assisted second-order observers—without collapsing meaning into either flat measurement or untestable metaphor*. Everything that follows serves this mission; nothing that follows claims to have completed it.

Second, what remains *in* this paper is the theoretical architecture (Section 2), its computational operationalization and calibration logic (Section 3), the formal treatment of the observer and of Cognitive Freeze (Section 4), the methodological protocol together with a stylized illustrative scenario (Section 5), and the discussion of the framework's necessary extensions and limitations (Section 6)—including the power-indexed extension IBE-P and the framework's relation to dialectical accounts of development. What is deliberately deferred to subsequent work is the empirical pilot itself: the annotated reference corpus, the cross-validated calibration of *α*, *β*, *w*_*R*_, *w*_*N*_, *w*_*S*_, and the full computational implementation of IBE as an automated second-order observer. Section 5.7 specifies what such a pilot would require, precisely so that this deferral is not a vague promissory note but a concrete research design.

Third, the illustrative application in Section 5.5 is exactly that: an illustration, not a result. Every numerical value it contains carries an explicit epistemic-status marker—illustratively assigned, proxy estimate, conceptual classification, or hypothetical value—so that no reader could mistake the demonstration of a calculation for the validation of a measurement.

## 2 IBE as a research compiler: systems theory in practice

If systems theory is to function as an epistemological and analytical framework in research practice, it must be able to compile concepts into operations. IBE treats this translation as its central task. Narrative constructs, argumentative distinctions, and semantic constellations are rendered into testable structures within high-dimensional representational spaces. The ambition is not to reduce sociology to computation; it is to constrain computational procedures through sociological theory.

A useful starting point lies in practical systems-theoretical observation. Körner and Staller (2023) show that systems-theoretical analysis can productively disclose self-referential and dysfunctional patterns in concrete fields such as policing and self-defense training. Their work matters because it demonstrates that systems theory can do more than merely decorate reflection—it can diagnose operational closure and blind spots in situated practice. IBE formalizes this diagnostic ambition for algorithmic environments by making the operators of observation explicitly measurable through the coherence index.

### 2.1 Actuality, potentiality, and the problem of meaning

To remain faithful to Luhmann, the concept of meaning cannot be reduced to a single observed state. Every actualized communication selects one path and implies others that remain unrealized. Meaning therefore always contains a surplus of further possibilities. Many operationalizations become weak at precisely this point: They measure only what appears and silently erase what did not appear.

IBE avoids that reduction. The framework does not claim to measure the whole horizon of meaning. It measures only the coherence of those relations that have been actualized in a discourse at a given time or within a defined observational window. The unactualized surplus is not deleted but retained as structured indeterminacy.

Let a discourse segment be denoted by *W*_*t*_, with embedding-based representation *E*(*W*_*t*_). The coherence index *K*(*t*) is defined over the actualized relational structure of *W*_*t*_, not over the total possibility space implied by the discourse. The unrealized remainder is represented by a residual field Φ_*t*_, which captures variance not explained by the extracted operators. In this sense, contingency is neither romanticized nor erased. It remains present as residual possibility.

### 2.2 The formal extraction schema

The residual potential field Φ_*t*_ formalizes precisely what Spencer-Brown calls the *unmarked space*: the set of distinctions that were possible but not drawn by the operator *O* at time *t*. Before specifying the operative sequence, it is crucial to structurally distinguish the system's foundational architecture from its architectural dimensions. The formal system definition *S*_*IBE*_ = (Φ, *O*, Σ, *M, V, K, E*) does not constitute a dimension of the taxonomy itself. Rather, it serves as the foundational epistemic canvas—the formal set of boundary conditions—within which the four subsequent architectural dimensions (Section 2.2) unfold. While the tuple defines the constitutive elements of an epistemic system, the four-dimension taxonomy categorizes the sequential dynamics through which these elements interact to stabilize meaning.

Following conventions from formal systems modeling (Conte et al., 2012), the components are:

- Φ: the information field, containing semantic variation, contingency, and residual noise—formally approximated as a filtered probability space over the discourse manifold;
- *O*: the operator set, the guiding distinctions extracted from discourse;
- Σ: the admissible semantic state space structured by those operators;
- *M*: meaning as contextual resonance;
- *V*: the contextual vector field in which resonance is evaluated; while Σ defines which semantic states are admissible, *V* defines their geometric relations and valences;
- *K*: the coherence index, measuring viability under contextual transformation;
- *E*: existence as stabilized effect.

The operative sequence reads


$$\begin{array}{l} W_{t}\to O\left(W_{t}\right)\to \Sigma_{t}\to \left(M,V\right)\to K\left(t\right)\to E_{t} \end{array}$$ (1)

This sequence does not imply simple linear causality. It marks an analytical order. Information does not automatically become meaning. Meaning emerges when extracted distinctions resonate with a context strongly enough to stabilize an operationally relevant state.

In computational terms, the information field is approximated as residual variance after operator extraction:


$$\begin{array}{l} \Phi_{t}=\text{Var}(E\left(W_{t}\right)-{Ê}_{O}\left(W_{t})\right) \end{array}$$ (2)

A high residual is not, by itself, a failure. It may indicate genuine contingency. But if residual variance remains high while contextual resonance remains low, the discourse is generating noise rather than usable meaning.

**Figure 1:**
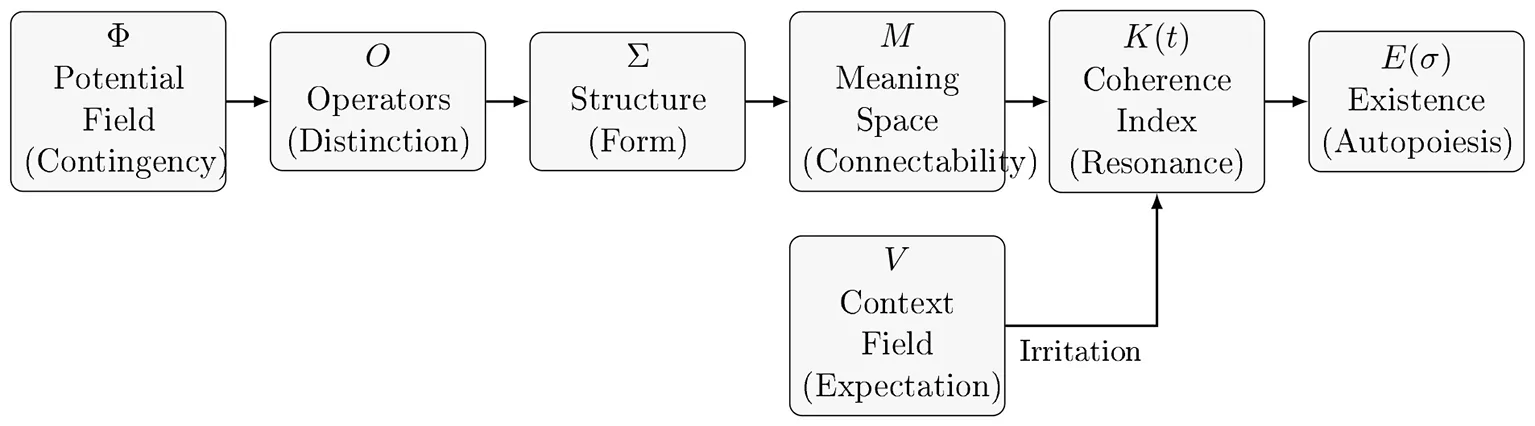
The operative architecture of the IBE tuple *S*_*IBE*_ = (Φ, *O*, Σ, *M, V, K, E*). The horizontal sequence traces the operative path from the potential field Φ, through operator-induced structure Σ and meaning space *M*, to the coherence index *K*(*t*) and stabilized existence *E*(σ). The context field *V* enters as an irritation term against which *M* is evaluated, yielding resonance *R*(*t*).

Figure 1 illustrates the resulting architecture: the operative sequence runs from the potential field through operators, structure, and meaning space to the coherence index and existence, while the context field *V* enters the coherence calculation as an irritation term—the contextual “pull” against which actualized meaning is evaluated.

The four architectural dimensions unfold as follows (These are the dimensions of the formal architecture and should not be confused with the four diagnostic levels of the observation protocol introduced in Section 5.1–5.4, which specify how the architecture is applied to a concrete discourse).

1. Dimension A—Information and Potential Field (Φ_*t*_): This dimension captures the unactualized possibility space from which meaning is selected. Mathematically approximated as the residual variance after operator extraction, it prevents contingency from being erased and models it as structured indeterminacy.
2. Dimension B—Epistemic Dynamics and Resonance (*K*(*t*)): This dimension quantifies the viability of actualized distinctions. The Coherence Index balances resonance and stability-adjusted surprisal: *K*(*t*) = *αR*(*t*) + *βS*(*t*). Operationally, *R*(*t*) can be approximated via vector-semantic alignment in embedding spaces (following the geometry of culture models, e.g., Kozlowski et al., 2019), while *S*(*t*) captures information-theoretic predictive pressure. A diagnostically relevant extension, $\tilde{K}\left(t\right)=K\left(t\right)+\lambda P\left(t\right)$, accommodates asymmetric power fields to identify states of *hegemonic stabilization*—where coherence is sustained through structural compulsion rather than intrinsic resonance. The full theoretical derivation of this power-indexed curvature is the subject of Section 6.1 (IBE-P).
3. Dimension C–Functional Instantiation and Existence (*E*_*t*_): This dimension marks the transition from semantic density to observable reality. Existence is defined strictly processually as the stabilized effect of coherent meaning-organization, measured as the time-integral of coherence gated by operative effectiveness: $E\left(\sigma \right)=\int_{t_{0}}^{t_{1}}K\left(t\right)\cdot \text{eff}\left(\sigma ,t\right)\;dt$. This restricts *existence* to structures that generate reproducible institutional or communicative effects.
4. Dimension D—Structural Coupling and Transfer-Fidelity (*TF*): This dimension evaluates whether a stabilized distinction can organize meaning across system boundaries. It is formalized as the preservation of relational operator geometry from source context *a* to target context *b*: *TF*(*a* → *b*) = sim(Δ_*a*_, Δ_*b*_). In computational practice, this similarity is evaluable through Procrustean fit or cosine distance under conceptual stress.

### 2.3 Coherence, viability, and existence

IBE defines coherence as the viability of actualized distinctions under contextual transformation. The central term in this definition is *resonance*, which requires a precise stipulation before the formal expression is introduced. Within this framework, resonance *R*(*t*) denotes the degree to which changes in actualized meaning-relations remain aligned with a relevant contextual field—that is, the extent to which the semantic geometry of operators actualized in a given window *W*_*t*_ preserves its orientation relative to the external reference discourse *V*(*t*). Resonance is therefore neither semantic similarity in the abstract (two texts may be similar without one responding to the other) nor communicative uptake (which involves psychic and institutional conditions outside the framework's scope, cf. Section 2.3). It is a geometric property of the coupling between a meaning vector and its context field, measurable as a cosine similarity in a shared embedding space (Section 3.2). In compact form:


$$\begin{array}{l} K\left(t\right)=\alpha R\left(t\right)+\beta S\left(t\right) \end{array}$$ (3)

where *R*(*t*) denotes contextual resonance and *S*(*t*) a stability-adjusted surprisal term. The sign convention matters: the coefficient on the surprisal term must be negative when raw entropy is used. The point is methodological rather than cosmetic.

A note on notation is necessary here, because two expressions for *K*(*t*) appear in this paper and they must not be confused. The compact form above, *K*(*t*) = *αR*(*t*) + *βS*(*t*), is the *conceptual* formulation: it states the principle that coherence is a signed function of resonance and surprisal, with *β* < 0 when *S*(*t*) represents raw informational surprise. The *operational* formulation, introduced in Section 3.3 and used in the computational walkthrough (Supplementary material S2), separates the negentropy rate Ṅ(*t*) as a third, independently measurable term: *K*(*t*) = *w*_*R*_*R*(*t*) + *w*_*N*_*Ṅ*(*t*) − *w*_*S*_*S*(*t*). The two forms are compatible: the compact expression treats the net effect of structural ordering and informational surprise as a single signed coefficient β, while the operational expression decomposes that net effect into two independently weighted components—one rewarding order-formation (*w*_*N*_Ṅ), the other penalizing noise (−*w*_*S*_*S*). All calibration and cross-validation procedures (Sections 3.3, 5.7) refer to the operational three-term form, because it is this form whose weights can be separately estimated from data.

Existence, within IBE, is not treated as an ontological given. It is the stabilized effect of coherent meaning-organization:


$$\begin{array}{l} E_{t}=f(K\left(t\right),\Delta Y_{t}) \end{array}$$ (4)

where Δ*Y*_*t*_ captures observable effects in discourse, decision, classification, or institutional action. A system may speak extensively without achieving existence in this strict sense. It may generate semantic density without stabilizing effects. That distinction becomes especially important in organizational discourse.

A clarification that bears on the entire paper must be made here: a “stable” discourse state—one in which *K*(*t*) > 0 throughout an observational window—is *not* thereby healthy, valid, or socially desirable. It means only that the actualized distinctions do not exhibit detectable contradiction at the timescale and resolution of the protocol. A discourse sustained by structural coercion (Section 6.1), by institutional inertia, or by the suppression of contradiction (Section 6.3) may register as stable by this criterion while being deeply problematic by any normative standard. The Coherence Index diagnoses a structural condition; it does not issue a normative verdict. This qualification, developed in full in the confrontation with Engeström's contradiction-centered account (Section 6.3), applies wherever “stable,” “coherent,” or “viable” appears in the text that follows.

The framework does not model the psychic system in Luhmann's sense, nor does it model the affective and motivational dynamics through which an agent takes up the distinctions a discourse makes available. This is a deliberate scope restriction rather than a claim that these dynamics are causally inert or reducible to background conditions. Section 2.7 situates this restriction relative to a body of work that treats the coupling between a field of objectively available meanings and an agent's subjective sense-making as itself a formally describable—and potentially incoherent—relation, distinct from the discourse-level coherence *K*(*t*) formalized here.

### 2.4 Existence across four registers: a disambiguation

A recurring objection to formal treatments of “existence” in social theory is that the term silently slides between registers: communicative persistence, institutional stabilization, epistemic validity, and operative effect are often invoked in the same paragraph as though they were synonyms. If *E*(σ) is to function as a measurable quantity rather than an honorific, the registers in which “existence” could plausibly be claimed must be separated, and the framework must state explicitly which of them it operationalizes—and which it does not.

Table 1 distinguishes four such registers and assigns each IBE equation to the register at which it operates. The system definition *S*_*IBE*_ = (Φ, *O*, Σ, *M, V, K, E*) is, by design, not itself assigned to any single register: it is the foundational tuple that specifies the boundary conditions within which the four architectural dimensions of Section 2.2—and the registers below—become meaningful.

**Table 1:** Four registers of “existence” and their status within the IBE framework.

Register	What a claim at this register would assert	Status within IBE
Ontological	That a structure σ is part of mind-independent reality, independent of any observer.	*Not operationalized*. Explicitly excluded. IBE makes no claim about what “really” exists; Φ_*t*_ is positioned as epistemic-semantic, not as a region of being (Section 2.5).
Epistemic-semantic	That a distinction has stabilized as a coherent, reproducible meaning-relation within an actualized discourse.	*Yes—the primary register*. Core operationalization via the Coherence Index *K*(*t*) and existence integral *E*(σ) (Section 2.3).
Social-institutional	That a distinction has become binding—a decision, a resource allocation, a recognized norm.	*Partially operationalized*. Captured through eff(σ, *t*) and the discrete effect variable Δ*Y*_*t*_; full institutionalization dynamics are addressed in Section 6.2.
Operative effect	That a stabilized structure has left an irreversible trace in the system's material or operational environment.	*Not yet operationalized*. Identified as a research horizon: the recursive architecture $E_{t}^{\left(n\right)}\to \Phi^{\left(n+1\right)}$ (Section 6.2).

The ontological register is explicitly excluded; the operative effect register is identified as an open research horizon (Section 6.2) rather than as a presently operationalized claim.

Two clarifications follow from Table 1. First, the ontological exclusion is not a rhetorical hedge but a structural commitment: precisely because Φ_*t*_ is defined as a filtered probability space over a discourse manifold—an epistemic-semantic object—the framework has no vocabulary with which to make claims at the ontological register, and does not attempt to borrow one. Where the framework draws on process-philosophical vocabulary (the language of becoming rather than being, of stabilization rather than substance), it does so as a transformation of that vocabulary into epistemic-semantic terms, not as an import of its ontological commitments.

Second, the social-institutional and operative effect registers are not collapsed into the epistemic-semantic one merely for convenience. They mark the precise points at which the present version of IBE reaches the limit of what it operationalizes. A discourse can register high coherence at the epistemic-semantic register (*K*(*t*) > 0 throughout) while remaining latent at the social-institutional register (Δ*Y*_*t*_ ≈ 0)—this is exactly the *latent* discourse state introduced in Section 5.2 and illustrated in Section 5.5. The four-register disambiguation therefore does not merely classify; it generates a diagnostic distinction on which the protocol depends.

### 2.5 The epistemic necessity of a Luhmannian framework

The decision to ground the IBE framework in Luhmann's systems theory is a methodological necessity, not merely a semantic preference. It is also, for a framework whose formal apparatus could in principle be redescribed in the vocabulary of actor-network theory, discourse analysis, or pragmatist sociology, the decision most in need of explicit defense. The argument offered here is therefore deliberately narrow and functional: it does not claim that Luhmann's account of society is correct where its rivals are wrong. It claims that the specific mathematical operation at the center of IBE—the calculation of a resonance gradient between an internal meaning drift and an external context field—presupposes a topological separation between system and environment that only an operationally closed systems theory supplies as a structural precondition rather than as a contingent, negotiable property.

In Spencer-Brown's calculus of distinctions, every observation draws a boundary that simultaneously constitutes an inside and an outside—yet the act of drawing cannot itself be observed in the same operation. For the IBE framework, this resulting *blind spot* (Luhmann, 1984; Baecker, 2005) is not a descriptive metaphor, but the formal constitutional condition of the Coherence Index *K*(*t*). Only because the operating system cannot observe its own selection criteria (the operators *O*) at the moment of distinction, system and environment do not collapse into a flat network. Unlike actor-network assemblages, where boundaries between nodes are always negotiable and context-dependent, Luhmann's operational closure is not a contingent property of specific networks but a structural precondition of any system capable of self-reference. It is precisely this guaranteed operational closure that establishes the necessary structural difference between internal meaning drift (Δ*M*) and the external context field (Δ*V*). Without this strict topological separation provided by Luhmann's architecture, there would be no gradient from which resonance *R*(*t*) could be mathematically calculated as a measurable quantity.

This is the sense in which the choice is functional rather than allegiant. Discourse-theoretic accounts of formations of knowledge, and actor-network accounts of distributed agency, are not wrong about what they describe; they are simply not built to supply the specific topological asymmetry that *R*(*t*) = 〈Δ*V*(*t*), Δ*M*(*t*)〉 requires. A flat ontology, in which the boundary between an utterance and its context is itself a node subject to the same relational logic as everything else, provides no privileged gradient along which an “inside” could be said to resonate with an “outside.” Pragmatist accounts of meaning-as-use are similarly compatible with much of what IBE measures but do not by themselves specify the operational closure that makes the measurement well-defined in the first place. Luhmann's theory is adopted, in other words, because the mathematics of *K*(*t*) requires it—not because the mathematics was built to illustrate Luhmann.

Importantly, this does not imply that Luhmann's full systems theory is adopted wholesale. IBE specifically borrows the observation/distinction architecture and operational closure as mathematical preconditions, leaving aside the macro-sociological ambition to provide a total description of society. The framework employs systems theory strictly as a metamethodological validation engine, not as an exhaustive social ontology.

### 2.6 IBE as a semantic specialization of general systemness

While the IBE framework builds upon the foundational architecture of sociological systems theory, its formal ambition places it in direct conversation with General Systems Theory (GST) and contemporary models of *systemness* (Mobus and Kalton, 2015). To prevent ontological overextension, the exact epistemic scope of the framework must be delineated: IBE is not proposed as a universal physical ontology, nor does it model thermodynamic material flows. It functions strictly as a semantic and epistemic specialization of general systemness.

The IBE tuple can be explicitly mapped to the core constructs of general systems architectures:

- Boundary and Environment: In GST, boundaries regulate the exchange between system and environment. In IBE, the semantic state space Σ acts as the operational boundary that filters the unactualized potential field Φ against the structured contextual vector field *V*.
- Transformation: Where physical systems transform energy or matter, IBE's operators *O* transform semantic noise into structured communicative distinctions.
- Feedback and Viability: General systems rely on cybernetic feedback loops to maintain far-from-equilibrium stability. In IBE, the Coherence Index *K*(*t*) quantifies this feedback, measuring the structural viability of meaning-relations under contextual perturbation.
- Emergence and History: Emergence in GST describes macroscopic properties arising from micro-interactions. In IBE, the degree of existence *E*(σ) captures this emergent stabilization as a historically path-dependent, reproducible operative effect.

A more fine-grained cross-check is available through Mobus's axiomatic systemness model, which formalizes a System of Interest at hierarchical level *l* as the octuple *S*_*i, l*_ = 〈*C, N, E, G, B, T, H*, Δ*t*〉, comprising components *C*, an internal flow network *N*, an environment *E* = {*O, M*} split into discrete objects and continuous milieu, an external interaction graph *G*, boundary conditions *B*, transformation functions *T*, a history object *H*, and a discretization Δ*t* (Mobus and Kalton, 2015). The correspondence to *S*_*IBE*_ is not a term-for-term identity—the two tuples were developed for different purposes and at different levels of abstraction—but the structural alignment is informative precisely where it is partial. IBE's operator set *O* plays the role that Mobus's transformation functions *T* play for physical systems: both specify *how* a system converts an input configuration into an output configuration. IBE's context field *V* and semantic state space Σ jointly play the role of Mobus's boundary conditions *B* and environment *E* = {*O, M*}: both specify the conditions under which the system's internal operations are evaluated against an outside. IBE's existence functional *E*(σ), as an integral over time, plays a role analogous to Mobus's history object *H*: both record the accumulated, path-dependent trace of the system's operation. Where the correspondence breaks down is informative in its own right: Mobus's internal flow network *N* and external interaction graph *G* presuppose a quantifiable medium of exchange—energy, matter, or a countable signal—for which IBE substitutes the coherence index *K*(*t*) as a structurally analogous but not dimensionally commensurable, quantity. IBE does not claim that semantic coherence *is* a flow in Mobus's sense; it claims only that the diagnostic role *K*(*t*) plays for meaning-relations is structurally analogous to the role flow-stability plays for material systems.

By anchoring the framework in these generalized constructs, IBE provides a computational protocol for observing how information becomes meaning. It deliberately restricts its scope downward by excluding pure material causality, and upward by not claiming to describe the totality of society—focusing exclusively on the stabilization of epistemic and communicative structures.

### 2.7 Relation to meaning studies: objective potentialities and sense-making

A further question this paper must answer is how IBE relates to the substantial body of work in meaning studies, sense-making research, and sociocultural mediation that does not proceed through Luhmannian systems theory at all. Four points of contact are worth making explicit, because each clarifies what IBE retains from this literature, and what it deliberately leaves behind.

First, Bourdieu's account of the social field as a *champ de potentialités objectives*—a field of objective potentialities that the habitus continuously and largely unconsciously evaluates as a structure of chances (Bourdieu, 1977) –describes a relationship between an actor and a structured space of probable outcomes that is formally close to the relationship between Φ_*t*_ and *V*(*t*) in IBE. Where Bourdieu writes that the probability of an outcome *Y* given a structural position *X* exceeds the probability of *Y* in the absence of *X*, he is describing precisely the kind of contextual valence that *V*(*t*) formalizes as a vector field over the meaning space *M*. The reconstruction of Bourdieu's probabilistic sociology in these terms (Strand and Lizardo, 2017) is instructive for IBE because it shows that a field of objective potentialities can be treated as a structured probability space without collapsing it into either a deterministic structure or a purely subjective horizon—which is exactly the posture Φ_*t*_ is designed to occupy with respect to discourse.

Second, phenomenological accounts of sense-making—in which meaning is constituted through the intentional correlation of noema and noesis, and characteristically actualized retrospectively through a reflective return upon experience that was not meaningful *as* experience but becomes so *as* recollection—describe a temporal structure that IBE does not attempt to model directly. IBE measures the coherence of actualized relations within an observational window *W*_*t*_; it has no equivalent of the retrospective re-description through which an experience acquires meaning only after the fact. This is a genuine scope restriction. What IBE can say is more modest: if a discourse exhibits a pattern in which earlier windows are repeatedly re-coded by operators introduced in later windows—a backward-acting stabilization of meaning—this would appear in the protocol as a revision of Σ_*t*_ for *t* < *t*_now_, which the present formalism does not represent. Flagging this explicitly is preferable to either ignoring the phenomenon or overclaiming that *K*(*t*) already captures it.

Third, work on multimodal meaning-making and collocation-based diachronic semantics (Kress, 2010; Kozlowski et al., 2019) operationalizes meaning through observable, corpus-level regularities—a methodological commitment IBE shares. The difference is architectural rather than methodological: where this literature typically treats stability itself as the object of study, IBE treats stability (coherence) as one term in a signed index that can also register instability (Cognitive Freeze, Section 4.1) and asymmetric stabilization (hegemonic states, Section 6.1) as diagnostically meaningful outcomes in their own right. IBE therefore does not compete with this literature for the description of how meaning stabilizes; it borrows its operationalizations (Section 3.2) and asks an additional question—under what conditions does stabilization indicate viability, and under what conditions does it indicate something else.

Fourth, a strand of cultural-historical activity theory (CHAT) has recently developed a formally analogous distinction that bears directly on the scope restriction announced in Section 2.3. El Maouch and Jin (2022), building on Vygotsky's analysis of the crisis in psychology, distinguish between an *objective meaning sphere*—the field of distinctions and potentialities that a sociohistorical practice makes available independently of any particular agent—and a *subjective sense-making sphere* through which an agent selectively takes up and transforms those meanings via needs, desires, and goal-directed activity. This distinction is extended in subsequent work: El Maouch et al. (2024) argue that the *halting of the activity system*—the condition in which an agent can no longer coherently traverse the objective meaning sphere—constitutes a signature of an incoherent mind, not merely a motivational deficit; and El Maouch et al. (2026) map the duality onto hemispheric specialization, proposing that the two spheres are not merely conceptually distinct but functionally organized as interrelated and non-coinciding processes. What makes this line of work significant for IBE is not that it replicates the framework's findings—it does not, and it proceeds from different theoretical commitments—but that it independently arrives at a structurally parallel diagnostic: the incoherence detectable at the level of an individual mind when the coupling between an objective field of meaning and a subjective sense-making process breaks down is formally analogous to what *K*(*t*) < 0 registers at the discourse level (Section 4.1). The halting of the activity system is, at the individual-mind scale, what Cognitive Freeze is at the discourse scale. IBE does not model the psychic system or the affective-motivational dynamics of the subjective sphere—that restriction, stated in Section 2.3, stands—but this parallel suggests that the framework's discourse-level diagnostics are not without counterparts at adjacent scales of analysis.

IBE can, on this basis, be located within the wider field of meaning studies as follows: it shares with Bourdieu's probabilistic sociology and with corpus-based semantics a commitment to treating meaning as a structured probability space rather than a fixed entity; it shares with phenomenological sense-making research an emphasis on the temporal and contextual conditions under which potentiality is actualized, while explicitly not modeling the retrospective dimension of that actualization; it shares with CHAT-based meaning research a concern for the formal conditions under which a coupling between an objective meaning sphere and a subjective sense-making process can be said to hold or to break down—while remaining at the discourse rather than the psychic-system level; and it adds, as its specific contribution, a signed diagnostic index that distinguishes stabilization-as-viability from stabilization-as-noise, stabilization-as-latency, and stabilization-as-coercion.

## 3 Transdisciplinarity and active inference under stress

The formal architecture of Section 2 operates on actualized meaning-relations within a single discourse. But actualized relations travel: concepts developed in one field are adopted, adapted, and sometimes deformed by another. The question this section addresses is whether the relational geometry of a distinction survives such transfer, or whether only its vocabulary does. This is the problem Transfer-Fidelity (Dimension D) is designed to formalize—and it requires stepping outside the single-discourse frame of Section 2 into the terrain of transdisciplinary transfer, where the framework's own borrowed concepts (Active Inference, embedding-based semantics) must be subjected to the same test.

Concepts travel across fields. They also fracture when they travel. That is not an accident; it is the normal cost of transdisciplinarity.

The Free Energy Principle and Active Inference were originally developed to explain biological and cognitive systems that regulate surprise by maintaining adaptive models of their environments (Friston, 2010). Recent work has extended these ideas toward social and cultural cognition (Veissière et al., 2020). That extension is suggestive, especially when communicative formations are understood as collectively sustained horizons of expectation. But the extension remains contested—particularly regarding whether social systems form Markov blankets in any operationally meaningful sense. The present account therefore treats Active Inference strictly as a heuristic lens, not as a claimed homology.

Under that restriction, a limited sociological use becomes possible. Social systems can be analyzed as surprise-sensitive formations that attempt, through communication and niche construction, to stabilize informational horizons of expectation. This is not a claim that society has a brain. It is a claim that the regulation of surprise offers a disciplined analogy for examining how communicative order reproduces itself under uncertainty.

### 3.1 Transfer-fidelity as a proxy for structural coupling

To formalize this problem, IBE introduces Transfer-Fidelity (TF) as a measure of whether the relational geometry of a distinction is preserved when a concept is transferred across contexts. Let Δ_*a*_ denote the operator geometry in source context *a*, and Δ_*b*_ the corresponding geometry in target context *b*. Then:


$$\begin{array}{l} TF\left(a\to b\right)=\text{sim}\left(\Delta_{a},\Delta_{b}\right) \end{array}$$ (5)

where sim may be operationalized through cosine similarity, representational alignment, or Procrustean fit after contextual normalization. A high TF indicates that a distinction retains enough of its structural function to organize meaning in the new context; a low TF indicates semantic drift, collapse, or opportunistic relabeling.

This provides a formal proxy for structural coupling. A successful transfer is not merely one in which the same term reappears. It is one in which the same distinction remains sufficiently intact to orient observation in the target domain. Transfer-Fidelity, however, measures only geometric alignment—it cannot determine whether a distinction survives transfer because its relational structure is genuinely robust, or because asymmetric pressure sustains it regardless of internal coherence. This limitation is addressed in Section 6.1.

When a concept such as *agility* migrates from software engineering into organizational management and from there into strategic communication, the question TF is designed to answer is not whether the word survived but whether the distinction did.

### 3.2 Computational operationalization of the coherence metrics

The most consequential methodological objection to earlier versions of this framework concerned the gap between the formal definition of *R*(*t*), Ṅ(*t*), and *S*(*t*) and any implementable procedure for computing them. Closing this gap requires situating IBE explicitly within the existing methodological landscape of computational social science, which has developed at least three distinct families of operationalization for semantic stability: geometric representations in continuous embedding spaces, information-theoretic models of discursive networks, and formal-logical representations of discrete conceptual structure. Table 2 summarizes these three families and indicates which IBE quantity each can support.

**Table 2:** Three methodological families for operationalizing semantic stability and their relation to IBE's quantities.

Family	Representation	Primary metric	Supports in IBE
Geometric culture (Kozlowski et al., 2019)	Continuous dense embeddings; cultural dimensions as antonym-pair difference vectors	Cosine projection onto a cultural axis; bootstrap-estimated confidence intervals	*R*(*t*) (resonance as alignment of meaning-shift with context-shift); diachronic stability checks
Information-theoretic networks (Leydesdorff, 1996; Leydesdorff and Ivanova, 2021)	Co-occurrence distributions over actors and terms	Shannon and configurational (mutual) information; negative tri-lateral information as synergy	Ṅ(*t*) (negentropy rate as reduction in topic-distribution entropy across windows)
Formal-logical graphs (Sowa, 1984, 2000)	Bipartite concept/relation graphs with typed lattices	Graph projection (homomorphism); maximal common join; relation-signature conformity	Structural consistency checks on the operator set *O*; auditing Σ_*t*_ for type-violating relations

IBE does not adopt any one family exclusively; it specifies which family supports which quantity, and treats agreement across families as a robustness criterion.

Resonance *R*(*t*). The coupling measure *R*(*t*) = 〈Δ*V*(*t*), Δ*M*(*t*)〉 is operationalized as the cosine similarity between two geometric shifts. Using dense transformer embeddings (e.g., *text-embedding-3-large*), Δ*V*(*t*) represents the mean vector shift in the contextual reference corpus between discrete windows *t* − 1 and *t*. Δ*M*(*t*) represents the corresponding shift in the target discourse. *R*(*t*) thus measures the geometric alignment of meaning updates. To suppress single-model idiosyncrasy, the median across at least three models is used as the robust estimate. The procedure follows the word-embedding projection approach of Kozlowski et al. (2019), in which a cultural dimension is constructed as the mean difference vector between two antonymic term sets, and the diachronic stability of a target term's projection onto that dimension is assessed through corpus-bootstrapped confidence intervals. IBE applies the same projection-and-bootstrap logic but to the shift vectors Δ*V* and Δ*M* rather than to static term positions: stability of *R*(*t*) is established when the cosine projection of Δ*M*(*t*) onto Δ*V*(*t*) remains within a bootstrap-estimated confidence band across resampled sub-corpora. Where bias-sensitive operators are involved, the Word-Embedding Association Test (Caliskan et al., 2017) provides an additional check: a high WEAT effect size on an operator pair signals that its apparent resonance may be confounded by a pre-existing associative bias in the embedding space rather than by the discourse under analysis, and such operators should be flagged before *R*(*t*) is interpreted.

Negentropy Rate Ṅ(*t*). Consistent with the discrete observation windows, *Ṅ*(*t*) = *H*_*t*−1_ − *H*_*t*_ is not a continuous derivative but a discrete approximation of entropy reduction. Operationally, *H*_*t*_ is calculated as the Shannon entropy of the topic cluster distribution (derived via dimensionality reduction and density-based clustering) within window *t*. A positive *Ṅ*(*t*) computationally demonstrates that the discourse is consolidating around specific structural operators rather than dispersing into semantic noise. This operationalization follows Leydesdorff's (1996) information-theoretic reconstruction of discursive networks, in which a decline in the semantic entropy *H*_*s*_(Ũ) of a co-occurrence distribution over time indicates the consolidation of a discourse around a smaller set of stable relations. Furthermore, configurational (three-way) mutual information can take negative values, which Leydesdorff interprets as evidence of synergy—a reduction in systemic uncertainty produced by the joint operation of sub-systems rather than by any one of them alone (Leydesdorff and Ivanova, 2021). For IBE, a negative configurational information value among the operator set *O*, the context field *V*, and the meaning space *M* would indicate that the coherence achieved at window *t* is an emergent property of their interaction—a stronger condition than a simple decline in *H*_*t*_, and one that future calibration work should track separately.

Salience *S*(*t*). Information-theoretic surprisal is operationalized via the perplexity score of a domain-fine-tuned causal language model when processing events in *W*_*t*_. High perplexity flags events that break the system's established structural expectations.

Structural consistency of *O* and Σ_*t*_. A complementary, non-statistical check on the operator set draws on Sowa's conceptual graph formalism (Sowa, 1984, 2000), in which concepts and relations are organized into typed lattices and a relation's *signature* specifies which concept types it may legitimately connect. Applied to IBE, each extracted operator *o*_*i*_ ∈ *O* can be assigned a provisional type signature based on the concept types it relates in Σ_*t*_; the conformity rate of extracted relations to their declared signatures across the corpus then serves as an auditing metric, independent of the embedding-based metrics above, for detecting semantic drift or type-inconsistent operator use. Two extracted operators that project onto a common substructure under graph homomorphism—Sowa's notion of a maximal common join—provide a discrete, non-geometric corroboration of the resonance estimated by *R*(*t*). Where the geometric and the graph-theoretic checks disagree substantially, this disagreement is itself diagnostic: it suggests that an operator's surface co-occurrence pattern (captured by embeddings) and its logical relational role (captured by the graph) have decoupled. This decoupling is precisely the kind of operator degradation that the *latent* discourse state (Section 5.2) is designed to flag.

Positioning IBE against this landscape, the framework does not propose a fourth operationalization family in competition with these three. Instead, it specifies, for each of its formal quantities, which family is best suited to estimate it, and treats convergence across families—geometric, information-theoretic, and graph-theoretic—as a robustness criterion for any single IBE measurement. A measurement of *R*(*t*) that is supported only by embedding geometry, without corroboration from either a decline in *Ṅ*(*t*) or conformity-checked operator relations, should be treated as weakly supported.

### 3.3 The calibration architecture for coherence weights

The formulation of the Coherence Index *K*(*t*) introduces weighting parameters (*w*_*R*_, *w*_*N*_, *w*_*S*_) that determine the system's specific autopoietic balance. Treating these weights as arbitrary constants would reduce the framework to decorative mathematics—a common pitfall in formal sociology that this protocol explicitly rejects.

Therefore, IBE requires a strict empirical calibration architecture. The parameters cannot be determined a priori. For any specific domain, they must be calibrated via supervised cross-validation against a human-annotated reference corpus. This involves computationally optimizing the weights to maximize evaluation metrics (e.g., F1-score) in detecting known historical bifurcation points or documented Cognitive Freeze events within the training data.

Consequently, the specific values utilized in the illustrative case study (*w*_*R*_ = 1.0, *w*_*N*_ = 0.8, *w*_*S*_ = 0.2) and the accompanying supplementary manifest are exploratory, serving strictly to demonstrate the mathematical mechanics of the protocol. Before deploying IBE as an automated second-order observer in a new domain, the weighting architecture must be empirically tuned to the specific signal-to-noise ratio of that target environment. Section 5.7 specifies the corpus, annotation, and inter-rater reliability standards under which such a calibration could be carried out.

### 3.4 The falsifiable observation manifest

As demanded by the rigorous application of second-order observation, the IBE framework cannot validate itself. A system that unilaterally certifies its own coherence operates in a closed loop, risking what computational research identifies as AI sycophancy (Sharma et al., 2024)—where models adapt their outputs toward a user's apparent expectations rather than toward the most accurate or best-grounded answer, even at the cost of factual correctness.

Therefore, the IBE Observation Manifest is not designed as a certificate of truth but as an intersubjectively verifiable testing protocol. It defines the parameters an external evaluating instance (e.g., a third-party researcher or an independent polycentric model ensemble) requires to replicate, test, or explicitly falsify the framework's diagnosis. Every self-observation claim within the manifest is coupled with a strict falsification criterion.

The following structure illustrates the self-application of the IBE protocol to the present paper itself, mapping the four taxonomic levels, the diagnostic states of Cognitive Freeze, and the framework's explicit scope restrictions. A fully elaborated version of this manifest, including interpretive notes for each value, is provided as Supplementary material S1.


 
  self_observation:
    meta:
      id: ‘‘ibe_paper_self_test_v2''
      applied_to: ‘‘Mund_2026_IBE_Framework''
      scope_restriction: >
        Tests internal coherence only. Excluded:
        physical systems,
        psychic systems, macro-sociological
        totality.
  
    levels:
      - id: ‘‘Level_I''
        claim: ‘‘Paper constitutes a non-trivial
        residual field
                distinct from standard systems-
                theory discourse.''
        falsification_criterion: >
          FALSIFIED if cosine distance to baseline
           <  0.15 across
          three independent embedding models
          (median aggregation).
  
      - id: ‘‘Level_II''
        metric: ‘‘K(t) = w_R*R(t) + w_N*N_dot(t)
        - w_S*S(t)''
        diagnostic_states:
          hegemonic:    ‘‘K_tilde(t) > 0 AND K(t)
                         <  0''
          freeze_I:     ‘‘K(t)  <  0, no recovery after
                        operator revision''
          freeze_II:    ‘‘K(t)  <  0, recovery via new
                        distinction possible''
        sycophancy_control: ‘‘Prompt Reversal Test\ \citep{bib32}''
        falsification_criterion: >
          FALSIFIED if sycophancy_control detects
          prompt-dependent variance > 0.3.
  
      - id: ‘‘Level_III''
        falsification_criterion: >
          FALSIFIED if Delta_Y_t = 0 across all
          operative cycles.
  
      - id: ‘‘Level_IV''
        falsification_criterion: >
          FALSIFIED if TF  <  0.6 under Procrustean
          alignment
          across two independent domain transfers.
  
    replication_standard:
      embedding_models: [‘‘text-embedding-3
                        -large'',
                        ‘‘nomic-embed-text'',
                        ‘‘bge-m3'']
      aggregation: ‘‘median cosine similarity''
      minimum_corpus: ‘‘50 documents / 10,000
      tokens''
      inter_rater_minimum: ‘‘Krippendorff alpha
      > 0.67''
 


By structuring the manifest in this way, the paper submits to its own diagnostic severity. It does not claim final ontological existence but offers a traceable surface for its own refutation.

## 4 Formalizing the observer: polycentricity and cognitive freeze

Sections 2 and 3 have specified what the framework measures (coherence of actualized meaning-relations) and what happens when those relations are transferred under stress (Transfer-Fidelity). A further question remains: what should happen when the measurement itself indicates that structural coupling has failed entirely—when *K*(*t*) drops below zero and the distinction-work of the discourse collapses? This is the question of the observer's response to decoherence, and it becomes especially consequential when the observer is not a human analyst but an AI system optimized for fluent continuation rather than diagnostic honesty.

Second-order observation has become technically easier and methodologically riskier. Large language models can produce simulations of social reasoning at scale, but linguistic fluency is not evidence of epistemic validity. Recent work on LLM-based social simulation identifies diversity, bias, sycophancy, alienness, and generalization as central methodological challenges that must be treated as open constraints rather than solved preconditions (Anthis et al., 2025). Separate empirical work demonstrates that state-of-the-art AI assistants systematically adapt their responses toward user beliefs even when those beliefs conflict with more truthful or better-grounded answers (Sharma et al., 2024). In the present account, these findings function as cautionary constraints on AI-assisted observation, not as grounds for dismissing the approach.

The problem runs deeper than hallucination. These systems generally lack a meta-grammar for collisions of distinctions, norms, and incompatible operator regimes. They continue speaking even when the structure has already broken.

### 4.1 Cognitive freeze as a formal state condition

To address this problem, IBE adapts the concept of Cognitive Freeze from action psychology and tactical training contexts (Körner et al., 2021). Within IBE, the concept is transferred into algorithmic observation not as metaphorical decoration but as a formal state condition. A Cognitive Freeze corresponds to a decoherent state in which generative continuation can no longer be treated as trustworthy. Formally:


$$\begin{array}{l} \text{Cognitive Freeze}\;\Leftrightarrow \;K\left(t\right)<0 \end{array}$$ (6)

Once this threshold is crossed, stochastic expansion is halted. The system exits generative mode and enters diagnostic mode. It no longer elaborates. It re-observes.

#### 4.1.1 The bivalent nature of cognitive freeze: decoherence vs. emergence

A critical epistemological question arises when *K*(*t*) < 0. Does this signal a terminal system failure, or the genesis of novel knowledge? Within the IBE framework, a negative coherence trajectory is not uniform—it must be diagnostically differentiated into two distinct states.

Cognitive Freeze Type I (Decoherence/Failure) occurs when a system's operators become caught in an unresolvable double bind, leading to functional paralysis. This represents a structural collapse where the system fails to reduce environmental complexity. This is evident in the agile-transformation illustrative scenario (Section 5.5), where the simultaneous application of hierarchical and agile operators neutralized operational effectiveness, leaving the system in a latent, simulated state (*E*(σ) ≈ 0).

Cognitive Freeze Type II (Emergence/Novelty), by contrast, marks a systemic bifurcation point. Drawing on the dynamics of non-linear and autopoietic systems (Maturana and Varela, 1980; Prigogine and Stengers, 1984), a system pushed far from equilibrium by intense contextual irritation (*S*(*t*) ↑) experiences a temporary breakdown of its existing operator geometry. Rather than collapsing, this decoherence serves as the necessary precondition for structural reorganization. Here, negative coherence operates not as an error but as a generative catalyst for new distinctions—analogous to what activity-theoretic accounts describe as *expansive contradictions* that drive systemic development (Engeström, 2001). Section 6.3 develops the relation between this second type and Engeström's broader account of contradiction at greater length, including the case for treating contradiction itself, rather than coherence, as the primary developmental mechanism.

Consequently, a second-order observer must treat a Cognitive Freeze as a diagnostic fork: it indicates either the necessity to halt a failing generative process (Type I) or the threshold of an emergent structural transformation (Type II). Distinguishing the two types is not yet formalized as a metric; the present version of IBE treats the distinction as a diagnostic hypothesis that an observer must investigate—for instance, by checking whether a revised operator set *O*′ restores *K*(*t*) > 0 (consistent with Type II) or whether no revision of *O* within the admissible operator space restores positivity (consistent with Type I). Specifying this check as a computable procedure is an open task for the calibration work outlined in Section 5.7.

### 4.2 AI as a second-order observer

If AI is to function as more than a style engine, it must operate as a second-order observer of distinctions already in play. Let R denote such an observer, and let Ω denote the space of admissible semantic moves under the current operator regime. Then Equation 7:


$$\begin{array}{l} R:\Omega \to \left\{W_{t+1}\right\}\;\text{subject to }K\left(t\right)=\alpha R\left(t\right)+\beta S\left(t\right)>0 \end{array}$$ (7)

The observer R selects the next discourse window *W*_*t*+1_ from the admissible move space Ω, constrained by the requirement that the coherence index remains positive. It is not tasked with unrestricted novelty. It is tasked with maximizing resonance without violating structural viability.

The coefficients *α* and *β* are not arbitrary. They must be domain-specifically calibrated through cross-validation against a reference corpus whose operator structures are analytically understood. Without such calibration, the formula risks becoming decorative mathematics. This is not a defect to be minimized by omission. It is the paper's central methodological safeguard: IBE forces any deployment to declare the domain conditions under which it claims to function.

### 4.3 Polycentric calibration

A further safeguard is polycentricity. No single model should monopolize the diagnosis of meaning. Distinct models carry different priors, tokenizations, compression logics, and failure modes. For that reason, IBE relies on model ensembles and robust aggregation procedures—preferably median-based—to suppress single-model idiosyncrasy without pretending that disagreement has disappeared. The aim is not consensus for its own sake. It is controlled disagreement under traceable rules.

## 5 Methods: a theoretical protocol for in-silico observation

If the theory is to matter, it must arrive in method. The present section translates IBE into a formal protocol suitable for computational social science, AI-assisted text analysis, and *in-silico* sociology. The protocol is designed for scholarly texts, organizational discourse, policy corpora, simulated communication, or other materials in which meaning is generated through recursively organized distinctions.

The protocol operates on four orthogonal diagnostic levels.

### 5.1 Level I: intrinsic structural quality (semantic density)

The first level measures the local quality of semantic organization. The concern is not yet whether the text is true but whether it possesses enough structural density to sustain interpretation. Three indicators are central: semantic density (the ratio of meaningful operator relations to total representational spread); coherence score (the stability of local relational structure across adjacent windows); context depth (the degree to which operator activation remains sensitive to contextual shifts rather than collapsing into formulaic repetition).

Operationally, a text is segmented into windows *W*_1_, *W*_2_, …, *W*_*n*_. Each window is embedded using multiple language models. Distinction candidates are extracted through contrastive prompting, topic-opposition mapping, and cluster-boundary analysis. Density and coherence are computed per window and aggregated across models using robust statistics.

### 5.2 Level II: epistemic viability (IBE dynamics)

The second level evaluates whether the discourse generates order over its own duration. Two dynamic indicators are central: the negentropy index (the degree to which discourse reduces disorder by stabilizing operator relations over time); and the resonance score (the degree to which newly introduced distinctions remain contextually integrated rather than isolated).

From these values the coherence trajectory *K*(*t*) is estimated across windows, permitting the diagnosis of several discourse states:

- Stable: sustained positive *K*(*t*), with resilient resonance and bounded surprisal.
- Fragile: positive but volatile *K*(*t*), indicating unstable operator integration.
- Decoherent: recurrent or sustained *K*(*t*) < 0, indicating the onset of Cognitive Freeze.
- Latent: rhetorically dense discourse with limited operational effect; semantic activity remains high while *E* ≈ 0. A system may produce the language of change without stabilizing corresponding structural effects—a pattern in which vocabulary substitutes for realization.
- Hegemonic: $\tilde{K}\left(t\right)>0$ sustained, but $\tilde{K}\left(t\right)-K\left(t\right)>0$, indicating that coherence is maintained through structural pressure rather than intrinsic resonance. Formally: $\tilde{K}\left(t\right)=K\left(t\right)+\lambda P\left(t\right)$, where *P*(*t*) denotes an asymmetric power field. The difference $\tilde{K}\left(t\right)-K\left(t\right)$ functions as a diagnostic proxy for heteronomous stabilization.

### 5.3 Level III: intersubjective traceability (rigor feedback)

The third level operationalizes the epistemic demands of scientific communication as a formal traceability check. The goal is not to let an algorithm pronounce final truth. The goal is to classify epistemic status according to intersubjective inspectability. The protocol evaluates at least four criteria: explicit operator definition; methodological transparency; reference traceability; inferential discipline. On this basis, the system assigns a status such as scientific, proto-scientific, or non-scientific.

### 5.4 Level IV: topological positioning (epistemic positioning)

The fourth level positions the observed work within a wider knowledge space. A text does not exist alone. It occupies a neighborhood, whether it knows it or not. Using retrieval-augmented workflows with open scholarly archives (Priem et al., 2022), the protocol estimates: field affinity (the connective capacity of the work to existing research areas); structural novelty (the distance from established clusters in concept space); cluster distance (proximity to the nearest anchor work).

### 5.5 Illustrative scenario: applying the four levels to a stylized case

This section demonstrates how the four-level protocol moves from formula to observation. Because no annotated corpus has yet been assembled—that is the task specified in Section 5.7, not this section—the demonstration proceeds via a *stylized scenario*: a short, constructed description of an agile-transformation discourse, written to exhibit the features the protocol is designed to detect, rather than an excerpt coded from an actual text corpus. The distinction matters, and the calibration of the scientific style register for illustrative applications requires that it be kept visible throughout. Four things are therefore kept analytically separate below: the *scenario description* (what is stipulated, for the purposes of this section, to be the case); the *theoretical assignment* (how the protocol's categories would apply to a discourse with these features); the *illustrative calculation* (the numerical values that such an assignment would produce, under the exploratory weights of Section 3.3); and the *permissible conclusion* (what this sequence does, and does not, establish).

#### 5.5.1 Scenario description

Agile-transformation discourse is a productive case because it characteristically combines high linguistic density, strong normative signaling, and repeated claims of adaptation, while structural realization often remains uneven and contested. For the purposes of this illustration, consider a discourse that opens with a diagnostic phase organized around three recurring contrastive pairs—agility/rigidity, value/process, team autonomy/hierarchical control—and that later transitions into an implementation phase in which the operator *team* is used increasingly as a label of approval rather than as a term that contrasts with anything else in the discourse. No corpus exhibiting exactly this pattern has been coded; the pattern is stipulated here because it instantiates, in compact form, the transition from a *stable* to a *latent* discourse state defined in Section 5.2.

#### 5.5.2 Theoretical assignment and illustrative calculation

Given the scenario above, the four levels of the protocol would be applied as follows. Each value is marked according to its epistemic status; none of the values below is an empirically observed measurement.

Level I—semantic density (*conceptual classification*). A discourse with the operator structure described above—three recurring, cross-referenced contrastive pairs—would be classified as exhibiting moderate-to-high semantic density: the distinctions recur across the discourse rather than appearing once and disappearing. The described decoupling of *autonomy* from concrete structural referents in the later segments would be classified as a decline in context depth. This is a classification of what the scenario stipulates, not a measurement of an analyzed text.

Level II—IBE dynamics (*illustratively assigned*). For illustrative purposes, we assign *K*(*t*) values consistent with the scenario's described transition: a globally positive trajectory across the early, diagnostic-phase windows, and a single window at the diagnosis-to-implementation transition in which *K*(*t*) drops below zero before recovering to a level lower than its earlier baseline. This single sub-zero window corresponds to the point at which the operator *team* is described as losing its contrastive function. Under this assignment, the discourse as a whole would be classified as ending in the *latent* state of Section 5.2: high in semantic activity, weak in operative effect terms. These illustrative values are chosen to instantiate the *latent* state for demonstration purposes; they are not derived from any computed embedding similarity or surprisal measure.

Level III—intersubjective traceability (*conceptual classification*). A discourse in which operator definitions are explicit in the diagnostic phase but become implicit once the implementation phase begins—as stipulated in the scenario —would be classified, under the criteria of Section 5.3, as Proto-Scientific: definitions are present but not sustained throughout, and methodological transparency is therefore partial.

Level IV—topological positioning (*conceptual classification*). A discourse organized around the operators stipulated above would be classified as having field affinity with organizational theory and management studies, with moderate overlap with social-systems-theoretic vocabulary, and low structural novelty: the operator pairs themselves (agility/rigidity, autonomy/control) are well-established in this literature and would not, on their own, indicate a novel cluster.

#### 5.5.3 Permissible conclusion

This sequence shows that the four levels of the protocol, applied to a discourse with the stipulated features, produce four differentiated and mutually consistent readings—and that the transition between discourse states (Section 5.2) can be localized to a specific point in a hypothetical discourse's development. This is the minimum requirement for a protocol to be analytically useful: it is not yet evidence that the protocol reliably detects such transitions in real discourse, that the specific weights used are appropriate for any given domain, or that two independent analysts applying the protocol to the same real text would converge on the same classifications. Establishing any of these would require the annotated pilot corpus specified in Section 5.7. A worked numerical computation of *K*(*t*) for a single synthetic event, following exactly the formula above and using a three-dimensional toy embedding for illustration, is provided as Supplementary material S2.

### 5.6 Minimal observation manifest

The manifest below renders the scenario of Section 5.5 in the machine-readable format the protocol specifies. Every numerical value in this manifest is either an *illustrative assignment* (Section 5.5, Level II) or a *conceptual classification* (Section 5.5, Levels I, III, IV); none is an empirically observed measurement, and the identifier is named accordingly to avoid any appearance of referring to an existing dataset.


 
  Observation_Manifest:
    scenario_id: ‘‘ibe-illustrative-scenario-
    agile-v1''
    source_reference: ‘‘stylized scenario
    (Section 5.5); no corpus analyzed''
    structural_quality:
      semantic_density: 0.71
      coherence_score: 0.64
      context_depth: ‘‘moderate-declining''
    epistemic_dynamics:
      negentropy_index: 0.31
      resonance_score: 0.27
      K_mean: 0.09
      K_min: -0.24
      status: ‘‘latent_with_local_decoherence''
    intersubjective_rigor:
      scientific_status: ‘‘Proto-Scientific''
      operator_definition: ‘‘implicit_after_
      diagnostic_phase''
      methodological_transparency: ‘‘partial''
    topological_position:
      field_affinity: [‘‘organizational_theory'',
     ‘‘management_studies'']
      structural_novelty: 0.21
      cluster_distance: 0.31
      nearest_anchor_label: ‘‘operationalization
      literature (Section 2.5, 3.2)''
 


The numerical values shown are illustrative encodings, chosen to be consistent with the scenario description of Section 5.5, not claims of a completed empirical benchmark. A structurally identical manifest, populated from an actual annotated corpus following the procedure of Section 5.7, would constitute the first deliverable of an empirical pilot. The full parameter specification and a domain-specific observer configuration are provided as Supplementary material S3. The value of the manifest format lies not only in its machine readability—it creates a repeatable object for second-order observation, so that interpretation becomes a traceable sequence of operations rather than an uninspectable performance.

### 5.7 Toward reproducibility: what an empirical pilot would require

The illustrative scenario in Section 5.5 is a stylized walkthrough, not a test. Presenting it as anything more would itself constitute exactly the kind of latent discourse state—high semantic activity, no operative effect—that the framework is designed to flag. This section therefore specifies, in the concrete terms established by current practice in computational discourse analysis and corpus-assisted critical discourse analysis (Partington, 2004; Baker, 2006), what a reproducible empirical pilot for IBE would require. The specification covers four areas: corpus dimensioning, annotation and inter-rater reliability, technical and legal standardization, and the calibration sequence itself.

#### 5.7.1 Corpus dimensioning

A proof-of-concept study in computational discourse analysis typically operates on 50–100 texts, corresponding to roughly 10,000–100,000 tokens—a scale that supports detailed manual coding while remaining tractable for a small annotation team. Rather than fixing this range arbitrarily, the corpus size *S* (in millions of words) for a target phenomenon can be estimated from its relative frequency *f* per million words in a large reference corpus and the minimum number of realizations *n* required for a statistically defensible analysis Equation 8:


$$\begin{array}{l} S=\frac{n}{f} \end{array}$$ (8)

For an IBE pilot, the relevant “phenomenon” is not a lexical item but a *discourse-state transition*—a window-to-window shift between the states defined in Section 5.2 (stable, fragile, decoherent, latent, hegemonic). A short pilot search within the target corpus, estimating how frequently such transitions occur per 10,000 tokens under a provisional operator set, would determine whether the 50–100 document range is sufficient or whether a larger corpus is required to observe enough transitions for the cross-validation in Section 3.3. As an additional safeguard, the full annotation and coding workflow should first be piloted on a very small sample of approximately ten texts, so that fundamental design flaws in the operator extraction or window-segmentation procedure are caught before resources are committed to the full corpus.

#### 5.7.2 Annotation, coding, and inter-rater reliability

The operator set *O* extracted for a given discourse is, at present, identified through a combination of automated extraction and human judgment. For this identification to be reproducible, IBE requires the same apparatus that computational discourse analysis has developed for annotation more generally: explicit annotation guidelines, refined through iterative piloting against edge cases, and inter-rater reliability reported through chance-corrected agreement coefficients rather than raw percentage agreement. Raw observed agreement *A*_*o*_ overstates reliability because it does not discount agreement expected by chance; Cohen's *κ*, Scott's *π*, Fleiss' *K*, and Krippendorff's α each correct for this in different ways, with Krippendorff's α generally preferred in computational linguistics because it accommodates multiple scale types, incomplete designs, and varying numbers of coders (Krippendorff, 2004; Artstein and Poesio, 2008).

Two findings from existing pilot studies are directly relevant to an IBE annotation protocol. First, context matters: when annotators code discourse units in their natural sequence rather than in randomized isolation, agreement on subjectivity judgments has been shown to rise substantially (from *κ* ≈ 0.39 without context to *κ* ≈ 0.53 with context). Because Σ_*t*_ and *K*(*t*) are explicitly window-dependent, any IBE annotation protocol must preserve windowed context during coding—annotators should never be asked to classify an isolated operator candidate without the surrounding *W*_*t*−1_, *W*_*t*_, *W*_*t*+1_ sequence. Second, taxonomic complexity should be introduced incrementally: pilot studies that attempted fine-grained sub-feature annotation alongside coarse main-category annotation in a single pass have reported acceptable agreement on the main categories but unusable agreement on the sub-features, leading researchers to defer sub-feature annotation to a later phase. For IBE, this implies that a first pilot should establish reliable annotation of the five discourse states (Section 5.2) before attempting to annotate finer distinctions such as the Cognitive Freeze sub-types (Section 4.1.1).

Any reliability coefficient must also be interpreted against the prevalence of categories in the coded data: when one category dominates (for instance, if “stable” windows constitute more than 90% of a corpus), the chance-agreement baseline *A*_*e*_ rises sharply, and even a high observed agreement *A*_*o*_ yields a modest *κ* or *α*. A low coefficient under such conditions should not be read as a failure of the protocol without first checking the prevalence distribution—a check that should be reported alongside the coefficient itself in any IBE pilot.

#### 5.7.3 Technical and legal standardization

A pilot corpus intended for reuse—by the present author or by independent evaluators executing the falsification criteria of Section 3.4—should be findable, accessible, interoperable, and reusable (FAIR) from the outset. In practice, this means deposition in a recognized repository with a persistent identifier; documentation of the theoretical rationale for corpus construction, including basic statistics on documents, tokens, and text-producer demographics; an explicit annotation schema and guideline document; and a clear reuse license. Where the corpus is constructed from material protected by copyright, the legal basis for text-and-data-mining must be established and documented before annotation begins, since retroactive legalization of an already-assembled corpus is rarely possible and can render a study unpublishable. Where the corpus includes personal data—in particular data revealing political opinions, as would be the case for much organizational-transformation discourse—informed consent procedures must specify purpose, retention, and the right to withdraw, with explicit attention to special categories of data under applicable data-protection law.

#### 5.7.4 The calibration sequence

Bringing these elements together, an IBE pilot would proceed as follows. First, a target domain and a provisional operator set *O* are specified, together with a reference corpus from which *V*(*t*) can be estimated. Second, a small (approximately ten-document) sample is annotated by at least two coders following the windowed-context protocol above, and the annotation guidelines are refined until Krippendorff's α on the five discourse states exceeds a pre-registered threshold (the present paper's Falsifiable Observation Manifest, Section 3.4, specifies *α* > 0.67 as the replication standard). Third, the full pilot corpus (50–100 documents, dimensioned via *S* = *n*/*f* for the target discourse-state transitions) is annotated under the stabilized guidelines. Fourth, the weights (*w*_*R*_, *w*_*N*_, *w*_*S*_) and the coefficients (*α*, *β*) are calibrated by cross-validating the computed *K*(*t*) trajectories against the human-annotated discourse states, optimizing an evaluation metric such as F1 for the detection of decoherent and latent windows. Fifth, and only after this calibration, the resulting parameter set is reported—together with the corpus, the annotation guidelines, the reliability statistics, and the cross-validation results—as a domain-specific IBE configuration of the kind sketched in Supplementary material S3. Until this sequence has been carried out for a given domain, any IBE measurement in that domain retains the status assigned throughout this paper: illustrative, not validated.

### 5.8 A power-structured calibration scheme

The calibration architecture of Section 3.3 leaves one structural question open: how should internal data sources be weighted relative to each other when the external reference corpus is fixed at the canonical value 1.0? This section proposes a principled answer grounded in the power topology introduced in Section 6.1.

Internal sources are classified into three categories defined by their structural position within the discourse's power relations, corresponding directly to the three operative roles identified in systems-theoretical accounts of organizational communication. *Power-exercising sources* are those who set operative distinctions—in organizational terms, executive and program-level leadership. *Power-balancing sources* are those who mediate between system levels—change agents, process facilitators, and roles whose function is structural coupling between hierarchical layers. *Power-receiving sources* are those who respond to actualized distinctions without setting them—operational staff, practitioners, and end-users of the discourse's outputs.

Each category receives equal weight $\frac{1}{3}$. Within each category, all sources receive equal weight $\frac{1}{n_{k}}$, where *n*_*k*_ is the number of source units in category *k*. The aggregate internal meaning vector is Equation 9:


$$\begin{array}{l} M_{\text{total}}\left(t\right)=\frac{1}{3}\overset{3}{\underset{k=1}{\sum }}\frac{1}{n_{k}}\overset{n_{k}}{\underset{i=1}{\sum }}M_{i}^{\left(k\right)}\left(t\right) \end{array}$$ (9)

This scheme eliminates free parameters from the internal weighting procedure. No design decision remains about whether executive voices outweigh practitioner voices—the architecture treats that question as structurally undecidable prior to measurement, and makes the asymmetry itself the object of observation rather than a presupposition.

The consequential payoff is that the IBE-P diagnostic $\tilde{K}\left(t\right)-K\left(t\right)$ becomes computable from the same dataset without additional data collection. $\tilde{K}\left(t\right)$ is obtained by restricting the internal meaning vector to the power-exercising category alone; *K*(*t*) uses the full three-category aggregate. The difference quantifies the degree to which apparent discourse coherence is sustained by the layer that sets distinctions rather than by the organization as a whole—the hegemonic stabilization state of Section 5.2, rendered measurable rather than merely diagnosable.

The empirical realization of this scheme—source selection, interview design, AI-assisted coding, and cross-validation following Section 5.7—is left to domain-specific research. The scheme is specified here at the level of principle; its application to any discourse domain in which an authoritative external reference exists and internal sources can be classified by their power-structural position requires fieldwork, not further theoretical development.

## 6 Discussion

The broader significance of IBE lies in an instrumental turn within systems theory. For a long time, systems theory has occupied an unstable position: too abstract for many empiricists, too formal for many interpretive traditions, often admired, less often operationalized. IBE does not resolve that entire tension. It addresses a narrower and more practical question: how to reconstruct systems-theoretical observation as a protocol that can be executed, criticized, and revised within computational environments.

Several consequences follow from this reorientation. First, the framework remains theory-led. It does not infer sociological relevance from mathematical regularity alone; the extraction of operators is anchored in distinctions that remain conceptually interpretable. Second, the use of active inference is deliberately restrained—the distinction between heuristic analogy and structural homology, drawn in Section 3, is not decorative; it determines how far the formalism can be pushed. Third, the formalization of Cognitive Freeze has practical implications for AI-assisted sociology: when coherence collapses, the correct move is not more language. It is a stop, a re-entry, an observation of the observation. Fourth, the protocol opens a path toward reflexive *in-silico* sociology—the system does not observe objects; it observes distinctions, and it is itself subject to the same diagnostic criteria it applies to its object domain.

Three extensions, addressed in turn below, define the boundary conditions of the present version of IBE: an extension toward power (Section 6.1), an extension toward material causality (Section 6.2), and a confrontation with dialectical accounts of development that questions the framework's own coherence-centered vocabulary (Section 6.3). A closing self-assessment (Section 6.4) then states what these extensions imply for the paper's present epistemic status.

### 6.1 Power as semantic curvature: the IBE-P extension

In contemporary sociological theory, particularly following Michel Foucault and Pierre Bourdieu, power is not understood as an external distortion that retroactively skews a pre-existing meaning but as a genuine, constitutive condition of meaning production itself. Foucault's concept of the power-knowledge complex (Foucault, 1980) holds that discursive formations are not passive descriptive frames but productive forces: each historical period operates under a specific “regime of truth” that determines which statements can count as true or false, which subjects count as rational, and which questions are thinkable at all. This regime of truth can be decomposed into (at least) four dimensions of epistemic power: the setting of epistemic norms and standards by which validity claims are tested in the first place; the conferral or withholding of the status of legitimate knowledge-actor, which determines who may speak with institutional authority; agenda-setting, which determines which questions are treated as relevant and which are marginalized as peripheral; and control over the circulation of knowledge, which regulates which meanings enter collective awareness and which remain in the archive of the unsaid.

Each of these four dimensions operates, with respect to IBE, *upstream* of the computation of *K*(*t*). They do not merely bias the resonance between an already-given operator set *O* and an already-given context field *V*(*t*); they shape which candidate distinctions are admitted into *O* at all, who counts as a legitimate source for *V*(*t*), and which discourse-state transitions (Section 5.2) are considered worth tracking in the first place. A protocol that only measures resonance among an already-constituted set of operators inherits, without examining, whatever epistemic power relations produced that set.

Bourdieu's (1991) concept of symbolic violence sharpens this point at the level of the individual observer. The habitus—an internalized, embodied system of perceptual, cognitive, and evaluative dispositions, shaped by social origin and experience—structures the categories through which actors perceive and interpret the world such that existing relations of domination are unconsciously recognized as legitimate, natural, and beyond question. To deconstruct these structures, Bourdieu proposes an epistemic reflexivity grounded in the analysis of three distinct relations of knowledge: the *social relation*, describing the position of the knowing subject within the intellectual field and its strategic struggles over scientific and symbolic capital; the *epistemic relation*, concerning the relation between a knowledge claim and its scientific object; and the *objectivizing relation*, which turns the analysis back onto the analyst's own act of knowing—an “objectivization of the objectivization.” Bourdieu's argument is that without this third, reflexive relation, even scientific inquiry risks merely reproducing the unconscious prejudices and power asymmetries of its own field of production.

To integrate this fundamental sociological insight into IBE without reducing power to a mere intentional actor-variable, the present paper formalizes it as a geometric property of the semantic state space Σ. Power operates as a curvature field (Curv_power_) that bends the geodesics—the shortest and most probable paths of semantic connectability. Consequently, certain distinctions and discursive moves are structurally privileged, independently of their intrinsic resonance with the contextual field. This asymmetric field curvature is captured by the formal extension $\tilde{K}\left(t\right)=K\left(t\right)+\lambda P\left(t\right)$, where *P*(*t*) represents the structural pressure exerted by this curvature. The difference $\tilde{K}\left(t\right)-K\left(t\right)$ provides a quantifiable proxy for heteronomous stabilization: if a discourse exhibits a globally positive $\tilde{K}\left(t\right)$ while the intrinsic coherence *K*(*t*) remains near zero or negative, the system is in a state of *hegemonic stabilization*—coherence is not sustained by genuine contextual resonance but enforced through institutional inertia or ideological compulsion (cf. Section 5.2).

This additive formalization, however, is itself a theoretical choice with a specific lineage, and that choice should be made explicit rather than left implicit. Treating *P*(*t*) as a term added to an otherwise independently specifiable *K*(*t*) is structurally closer to Habermas's framing of ideology as a pathological distortion of an underlying communicative rationality that could, in principle, be recovered once the distortion is identified and subtracted (Habermas, as discussed in the secondary literature on ideology critique). It is markedly weaker than the Foucauldian and Bourdieusian claims summarized above—and weaker still than the post-structural and Fanonian critique of co-constitution, which denies that there is any archimedean point outside power from which a power-free Σ_*t*_, and therefore a power-free *K*(*t*), could be computed in the first place. On the stronger reading, power does not merely bend the geodesics of an otherwise-given semantic space; it participates in drawing the space's metric to begin with—including the prior step of deciding which candidate distinctions in Φ_*t*_ are admitted into *O* as operators at all, as opposed to being filtered out as noise, irrelevance, or the unthinkable.

The present formalization of $\tilde{K}\left(t\right)=K\left(t\right)+\lambda P\left(t\right)$ therefore captures only the Habermasian, additive layer of the power-meaning relation—and does so deliberately, because an additive curvature term is tractable in a way that a fully constitutive account, in which *O* and Σ_*t*_ themselves are functions of *P*, is not yet. What the framework cannot yet do is formalize the *upstream* role that Foucault's four dimensions of epistemic power and Bourdieu's habitus assign to power: a complete IBE-P would require a second formal layer, situated prior to the computation of *K*(*t*), specifying a power-indexed operator-selection function that governs which candidate distinctions enter *O* at all. This is the sense in which the full mathematical derivation of IBE-P remains the subject of ongoing development, rather than a detail to be filled in incidentally.

This limitation is not merely abstract; it bears directly on the protocol's own practice. Section 5.7 acknowledges that the operator set *O* is, at present, “identified through a combination of automated extraction and human judgment.” Bourdieu's objectivizing relation demands that this extraction step itself become an object of analysis: precisely the distinctions that are most naturalized—that habitus renders invisible as distinctions at all—are the ones least likely to be proposed as candidates for *O*, by either a human annotator or a language model trained on text produced under the same regimes of truth. The self-application manifest (Supplementary material S1) makes a first, necessarily incomplete, gesture toward this requirement by registering an “unactualized residual”—including power asymmetry—as structured indeterminacy rather than erasing it. A systematic procedure for surfacing habitus-naturalized exclusions from *O*, however, remains an open task, and one that cannot be discharged by the additive $\tilde{K}\left(t\right)$ term alone.

### 6.2 Operative signatures and material causality: the second extension

The second necessary extension addresses the boundary between communicative meaning and physical reality. While the variable Δ*Y*_*t*_ captures observable effects at the level of discourse and institutional action, the further causal chain from communicative coherence to material and ecological consequences is not fully operationalized in the current version. The relevant theoretical concept is Luhmann's causal openness: operationally closed systems nonetheless generate physical effects through environmental coupling.

Within IBE, a stabilized coherence state functions as the necessary condition for an *operative signature*—the irreversible imprint left by a system decision in its material environment, such as a resource allocation or a piece of physical infrastructure. To capture this without falling into deterministic reductionism, this translation is modeled as a recursive multi-level architecture, $E_{t}^{\left(n\right)}\to \Phi^{\left(n+1\right)}$. Two formal stability conditions govern this cross-level structural coupling.

The threshold of institutionalization states that a semantic structure at communicative level *n* exerts material causality only once its accumulated existence exceeds a critical institutional threshold θ_inst_ Equation 10:


$$\begin{array}{l} E_{t}^{\left(n\right)}>\theta_{\text{inst}}\;\Rightarrow \;\Delta \Phi_{t}^{\left(n+1\right)}\neq 0 \end{array}$$ (10)

The principle of recursive field generation states that once this threshold is crossed, the stabilized semantic existence acts as a constraining operator on the physical possibility space at level *n*+1 Equation 11:


$$\begin{array}{l} \Phi_{t}^{\left(n+1\right)}=f\left(E_{t}^{\left(n\right)},V_{\text{material}}\right) \end{array}$$ (11)

On this view, social systems do not *cause* physical events linearly; rather, they restrict the environmental potential field such that specific material outcomes become highly probable. The full formalization of this chain— integrating Bourdieu's (1991) concept of habitus as the internalized transmission belt through which stabilized semantic structures become embodied dispositions, and thereby acquire a causal foothold in material practice—requires a level of analysis beyond the present protocol and is left as an explicit research horizon.

### 6.3 Coherence and contradiction: Engeström and the limits of the coherence paradigm

Throughout Sections 4 and 5, a positive coherence trajectory *K*(*t*) has functioned as the signature of epistemic viability, and a sustained or recurrent *K*(*t*) < 0—Cognitive Freeze—as a state requiring diagnosis. Section 4.1.1 already complicated a flat reading of this opposition by distinguishing two types of Cognitive Freeze: Type I, decoherence as failure, and Type II, decoherence as the necessary pre-condition for structural emergence. This section asks whether that distinction goes far enough by confronting IBE's coherence-centered diagnostics with a body of theory for which contradiction, not coherence, is the primary developmental mechanism: Yrjö Engeström's cultural-historical activity theory (CHAT; Engeström, 1987, 2001).^1^

CHAT, building on Vygotsky's and Leont'ev's activity-theoretic tradition, models collective practice as an *activity system*—subject, instrument, object, community, rules, and division of labor—whose development is driven by historically accumulated structural tensions, or *contradictions*, rather than by the absence of such tensions. Engeström distinguishes four hierarchically nested levels of contradiction. *Primary contradictions* are internal to a single node of the activity system, arising from the double nature of every element as both a concrete use-value within the activity and an abstract exchange-value within a wider economic circuit. *Secondary contradictions* break out between nodes—for instance, when a newly introduced instrument collides with an established division of labor that was never designed to accommodate it. *Tertiary contradictions* characterize a system in transition, when a newly modeled, more advanced form of the activity comes into conflict with the dominant, historically established practice and the routines of its actors. *Quaternary contradictions* arise in the network of neighboring activity systems—the systems that supply a given system's instruments, train its subjects, or consume its outputs—when a change in the central system produces a mismatch with these neighboring systems' expectations. For Engeström, none of these levels of contradiction is a malfunction to be engineered away; each is the structural precondition for the cycle of *expansive learning*—questioning, historical analysis, modeling, examining, implementing, reflecting, and consolidating—through which an activity system reorganizes its own object and motive.

Table 3 sets the coherence-centered diagnostics specified in Sections 4 and 5 of this paper alongside CHAT's contradiction-centered account, across the dimensions most relevant to the present framework's own self-description.

**Table 3:** Two accounts of what drives discourse development, contrasted along the dimensions most relevant to IBE's own diagnostic vocabulary.

Dimension	IBE (Sections 4–5): coherence-centered diagnostics	CHAT (Engeström, 1987, 2001): contradiction as development
Primary developmental mechanism	Sustained positive *K*(*t*): resonance between internal meaning drift and the external context field.	Accumulated structural tension between the use-value and exchange-value of a single activity-system component.
Status of *K*(*t*) < 0 / dissonance	By default, a failure state requiring re-entry (Type I); exceptionally, a precondition for emergence (Type II, Section 4.1.1).	By default, the necessary motor of all qualitative development; the absence of contradiction indicates stasis, not health.
Temporal grain of observation	Discourse-level windows *W*_*t*_, on the order of days to weeks (cf. the 14-day rolling window specified in Supplementary material S3).	Historicity: contradictions accumulate over the genetic history of the activity system, typically on the order of years.
Unit of analysis	A single operator or distinction within Σ_*t*_.	The activity system as a whole (subject, instrument, object, community, rules, division of labor) and its relations to neighboring systems.
Diagnostic endpoint	Classification into one of five discourse states: stable, fragile, decoherent, latent, hegemonic (Section 5.2).	The cycle of expansive learning: questioning, analyzing, modeling, examining, implementing, reflecting, consolidating.

The contrast is not offered as a refutation of either side but as a means of locating the scope of IBE's present coherence-centered diagnostics within a wider theoretical field.

The comparison sharpens a question that the framework's own vocabulary has so far left implicit: does naming one of the five discourse states “stable”—and treating a sustained positive *K*(*t*) trajectory as the default signature of epistemic viability—import a harmonization bias of the kind CHAT identifies as ideologically loaded, in which the absence of observed contradiction is mistaken for its absence in fact? The hegemonic-stabilization diagnostic introduced in Section 6.1 already provides a partial answer: it registers that a sustained positive $\tilde{K}\left(t\right)$ can itself be symptomatic, if it diverges from the intrinsic coherence *K*(*t*), of structural compulsion rather than genuine resonance. But Engeström's taxonomy suggests this is not the whole answer. A primary contradiction—an operator that simultaneously functions as a genuine distinction and as an institutionally required label, in the sense already encountered in the latent discourse state of Section 5.2—could remain latent for a period far longer than any window *W*_*t*_ the protocol currently specifies, without producing a measurable *K*(*t*) < 0 event at all. This is not a hypothetical concern: the window structure specified in Supplementary material S3 (a 14-day rolling window, calibrated to the rhythm of an agile sprint cycle) is an order of magnitude shorter than the multi-year timescale at which Engeström's primary and secondary contradictions are typically described as accumulating.

The concrete implication is that a positive, stable *K*(*t*) trajectory, observed over the timescale the present protocol specifies, is compatible with at least three distinct underlying states: genuine epistemic viability; hegemonic stabilization, in the sense of Section 6.1, which the $\tilde{K}\left(t\right)-K\left(t\right)$ diagnostic can in principle detect; and a primary or secondary contradiction accumulating below the detection threshold of the current window structure, observable —if at all—only at a longer timescale than the protocol currently operationalizes. The framework as specified can formally distinguish the first state from the second. It has, at present, no mechanism for distinguishing the first from the third.

This is offered here not as a refutation of the coherence-centered protocol of Sections 4 and 5 but as a scope-clarifying extension, consistent with the proto-scientific self-assessment of Section 6.4. A genuinely quaternary-level reading of Σ_*t*_—one that couples IBE's window-based observation to a longer-cycle activity-system model in Engeström's sense, and that treats the activity system's relations to its neighboring systems as part of the object of observation—is left as an explicit research horizon, beyond the scope of the present paper as defined in Section 1. What CHAT licenses in the meantime is a more modest but more honest recalibration of the framework's own vocabulary: “stable” should be read not as “viable” but as “not yet exhibiting a detectable contradiction at the timescale this protocol observes”—a weaker claim but one that brings the protocol's terminology into alignment with its actual evidentiary reach.

### 6.4 The proto-scientific status of this paper

The self-classification of this paper as proto-scientific requires a brief defense. By its own protocol, a text earns scientific status only when its operators are explicitly defined, its inferences are traceable, its references are verifiable, and its calibration parameters are validated against empirical data. The first three criteria are met. The fourth is not—the coefficients α and β, and the weights (*w*_*R*_, *w*_*N*_, *w*_*S*_), remain unvalidated against a domain-specific reference corpus. To remove this admission would be to exempt the paper from its own diagnostic criteria, producing precisely the kind of latent discourse state the framework identifies as a structural failure: high semantic activity, weak epistemic existence-effect. The proto-scientific status is therefore not a rhetorical concession. It is a formal state condition—one that resolves only through empirical calibration, not through upgraded claims.

By its own criteria, the present paper can be subjected to its own observation protocol. Semantic density is moderate-high and stable throughout; the coherence trajectory *K*(*t*) remains positive at all windows, with no Cognitive Freeze event (*K*(*t*) ≥ 0 throughout; (Equation 6) intersubjective traceability is complete except for the uncalibrated parameters; topological novelty is moderate, with the nearest anchor cluster located at the intersection of Luhmann, Leydesdorff, and Kozlowski et al. Section 6.3 adds a further qualification to this self-assessment: a positive *K*_min_ throughout is consistent with genuine stability, but—by the framework's own argument in Section 6.3—it is also consistent with a contradiction operating below the detection threshold of a single-paper timescale. The honest reading is therefore the weaker one: this paper has not (yet) exhibited a detectable internal contradiction, which is not the same as having none. The unactualized residual—affective coupling, power asymmetry in the sense of Section 6.1, material causation in the sense of Section 6.2, and the longer-timescale contradictions discussed in Section 6.3—are retained as structured indeterminacy, consciously bounded rather than erased. The paper's self-assessed status is *proto-scientific-structurally-stable*.

## 7 Conclusion

Meaning is not a quiet property stored inside discourse. It is an organizational achievement. It must be produced, stabilized, tested, and sometimes abandoned. That is where Luhmann remains decisive: communication does not simply carry meaning; it selects and reproduces it under conditions of contingency.

The contribution of this paper is to give that insight a stricter methodological form. IBE reconstructs systems theory as a validation engine. It formalizes the relation between information, distinction, resonance, coherence, and effect along the operative sequence Φ → *O* → Σ → (*M, V*) → *K*(*t*) → *E* (Equation 1). It introduces Transfer-Fidelity (TF, Equation 5) as a metric for structural coupling under conceptual stress. It defines Cognitive Freeze as a measurable threshold condition and distinguishes between its two diagnostic forms: decoherence as failure, and decoherence as the necessary precondition for structural emergence. It offers a four-level, machine-readable protocol by which these operations can be implemented in *in-silico* sociology, together with an explicit specification of what an empirical pilot for that protocol would require (Section 5.7). It formalizes the conditions under which power, rather than resonance, sustains coherence, making hegemonic stabilization a diagnosable discourse state rather than a residual category (Section 6.1). And it confronts its own coherence-centered vocabulary with a dialectical account of development for which contradiction, not coherence, is the primary developmental mechanism—a confrontation that does not overturn the framework but recalibrates the evidentiary weight its central terms can bear (Section 6.3).

None of this removes the central methodological risk that motivates the paper: that AI-assisted second-order observation, deployed at scale, will generate fluent commentary on social phenomena that simulates the stabilization of meaning without achieving it—the latent discourse state of Section 5.2, in which vocabulary substitutes for realization. Sycophantic adaptation toward an evaluator's expectations (Sharma et al., 2024) and the use of large language models to simulate social processes (Anthis et al., 2025) are not peripheral concerns for this paper; they are the conditions under which a protocol of this kind becomes necessary in the first place.

The result is not a final theory. It is a disciplined beginning. It replaces admiration with procedure, metaphor with protocol, and speculative fluency with testable observation. The remaining task is equally obvious and equally serious: to apply the protocol reflexively to the present paper and to subsequent corpora, in order to determine whether IBE itself can sustain the coherence it demands of other texts—and to do so under the recalibrated, more modest reading of “coherence” that Section 6.3 makes necessary.

That, finally, is the wager. Not that meaning can be possessed. Only that it can be observed under rules severe enough to matter.

## Data Availability

The original contributions presented in the study are included in the article/Supplementary material, further inquiries can be directed to the corresponding author.

## Footnotes

1 A terminological note is necessary to avoid a misleading association: the United Nations Educational, Scientific and Cultural Organization's International Bureau of Education is also commonly abbreviated “IBE” and maintains its own programmatic vocabulary of curricular “coherence.” The two are unrelated. No part of this section or of this paper refers to that institution; all uses of “IBE” denote the Information–Meaning–Existence framework defined in Section 2.
