# Supplementary material for: From information to existence: the Information–Meaning–Existence (IBE) framework as a meta-method for second-order observation
Source: Front Sociol. 2026 Sep 4;11:1871301. doi: 10.3389/fsoc.2026.1871301 (PMC13587620; doi:10.3389/fsoc.2026.1871301)
Supplement: Supplementary file 1 [file Supplementary_file_1.docx]

**Supplementary Material S1**

**Reflexive Self-Application of the IBE Observation Protocol**

Mund, H. (2026). From Information to Existence: The Information–Meaning–Existence (IBE) Framework as a Meta-Method for Second-Order Observation. Frontiers in Sociology 11:1871301.

DOI: 10.3389/fsoc.2026.1871301

This manifest applies the four-level IBE observation protocol (Section 5) to the present paper. It operationalizes the reflexive wager stated in the Conclusion and constitutes the paper's second-order self-observation. Values are author-assigned and subject to independent audit.

```
━━━━━━━━━━━━━━━━━━━━━━━━━━━━━━━━━━━━━━━━━━━━━━━━━━━━━━━━━━━━━━━━━━━━━━━━

Observation_Manifest:
  paper_id: "mund-ibev7-self-application"
  source_reference: "Mund (2026), From Information to Existence"
  doi: "10.3389/fsoc.2026.1871301"

  structural_quality:
    semantic_density: 0.74
    coherence_score: 0.71
    context_depth: "stable-with-deliberate-open-residual"

  epistemic_dynamics:
    negentropy_index: 0.44
    resonance_score: 0.61
    K_mean: 0.38
    K_min: 0.09
    status: "proto-scientific-structurally-stable"

  intersubjective_rigor:
    scientific_status: "Proto-Scientific"
    operator_definition: "explicit-throughout"
    methodological_transparency: "complete-except-calibration"

  topological_position:
    field_affinity:
      - "sociological_systems_theory"
      - "computational_social_science"
      - "AI-assisted_text_analysis"
    structural_novelty: 0.61
    cluster_distance: 0.44
    nearest_anchor_label: "Leydesdorff (1996), Kozlowski et al. (2019)"
    anchor_note: >
      The nearest anchor is not Luhmann directly but the operationalization
      literature — reflecting that IBE positions itself as a methodological
      extension, not a theoretical commentary.

  self_application_note: >
    The paper's K_min remains positive throughout, indicating no Cognitive
    Freeze event. The proto-scientific status reflects uncalibrated alpha/beta,
    not structural incoherence. The unactualized residual (affective coupling,
    power asymmetry, material causation) is retained as structured
    indeterminacy — not erased, but consciously bounded.

━━━━━━━━━━━━━━━━━━━━━━━━━━━━━━━━━━━━━━━━━━━━━━━━━━━━━━━━━━━━━━━━━━━━━━━━
```

**Interpretive Notes**

**Level I — Structural Quality**

semantic_density: 0.74. Operator relations are consistently maintained across all sections. The core tuple (Φ, O, Σ, M, V, K, E) recurs as an active referential structure, not decorative notation.

coherence_score: 0.71. Local relational structure remains stable across adjacent windows. No section introduces operators that are subsequently abandoned.

context_depth: "stable-with-deliberate-open-residual". Operator activation remains sensitive to contextual shifts throughout. The open residual is not a failure of context-sensitivity but a theoretical commitment: contingency is retained as structured indeterminacy (Φ_t), not collapsed into explained variance.

**Level II — Epistemic Dynamics**

negentropy_index: 0.44. Discourse reduces disorder by stabilizing operator relations over time. The operative sequence (Φ → O → Σ → (M,V) → K(t) → E_t) is introduced early and maintained as an organizing structure.

resonance_score: 0.61. Newly introduced distinctions (Transfer-Fidelity, Cognitive Freeze, IBE-P) remain contextually integrated rather than isolated. Each extension is anchored to the core tuple before being elaborated.

K_mean: 0.38 / K_min: 0.09. The coherence trajectory remains positive at all windows. No Cognitive Freeze event occurs. The paper does not continue generating language after structural breakdown — because no structural breakdown occurs.

status: "proto-scientific-structurally-stable". Structural stability is confirmed. Proto-scientific status reflects the uncalibrated state of α and β, not incoherence.

**Level III — Intersubjective Rigor**

scientific_status: "Proto-Scientific". Criteria met: explicit operator definition, methodological transparency, reference traceability, inferential discipline. Criterion not yet met: empirical calibration of α and β against a domain-specific reference corpus.

operator_definition: "explicit-throughout". All seven components of S_IBE are defined in Section 2.2 and remain consistently applied. No operator becomes implicit.

methodological_transparency: "complete-except-calibration". The protocol is fully specified. The exception is the calibration of the weighting coefficients α and β, acknowledged explicitly in Section 6 as the paper's open methodological task.

**Level IV — Topological Position**

field_affinity: Primary: sociological systems theory. Secondary: computational social science, AI-assisted text analysis. The paper operates at the intersection of these three fields without collapsing into any one of them.

structural_novelty: 0.61. Moderate-to-high. The contribution is not a new vocabulary but a new instrument: a validation architecture for systems-theoretical observation. This displaces the paper from pure theoretical commentary without placing it in the empirical methods literature.

cluster_distance: 0.44. The nearest anchor cluster is the operationalization literature (Leydesdorff, Kozlowski et al.), not Luhmann's primary texts. This is a finding, not an oversight: IBE is positioned as a methodological extension, not a theoretical commentary on Luhmann.

━━━━━━━━━━━━━━━━━━━━━━━━━━━━━━━━━━━━━━━━━━━━━━━━━━━━━━━━━━━━━━━━━━━━━━━━

**Closing Note**

This manifest does not claim to have won the wager stated in the Conclusion. It claims to have operationalized it — to have made the self-application observable, traceable, and open to independent revision.

The wager is not resolved by a positive K_min. It is resolved only when α and β are calibrated against an empirical reference corpus and the protocol is applied to a corpus beyond the paper itself.

Until then: proto-scientific-structurally-stable is the honest status. And honest status is the minimum condition for science to begin.

━━━━━━━━━━━━━━━━━━━━━━━━━━━━━━━━━━━━━━━━━━━━━━━━━━━━━━━━━━━━━━━━━━━━━━━━

*Mund (2026) — Supplementary Material S1 — IBE Self-Application Manifest*
