# Supplementary material for: From information to existence: the Information–Meaning–Existence (IBE) framework as a meta-method for second-order observation
Source: Front Sociol. 2026 Sep 4;11:1871301. doi: 10.3389/fsoc.2026.1871301 (PMC13587620; doi:10.3389/fsoc.2026.1871301)
Supplement: Supplementary file 2 [file Supplementary_file_2.docx]

**Supplementary Material S2**

**Computational Walkthrough: K(t) for a Synthetic 3D Event**

Mund, H. (2026). From Information to Existence. Frontiers in Sociology 11:1871301. DOI: 10.3389/fsoc.2026.1871301

This supplement reproduces the computation of the Coherence Index K(t) for a single synthetic event, using a three-dimensional toy embedding space for illustrative purposes. All values are hypothetical; the calculation demonstrates the mechanical application of the formulas in Section 3.2 and is not an empirical measurement.

━━━━━━━━━━━━━━━━━━━━━━━━━━━━━━━━━━━━━━━━━━━━━━━━━━━━━━━━━━━━━━━━━━━━━━━━

**Step 1: Define Vectors**

Meaning Vector m(t) — representing the discourse window W_t ("Directive"):

m(t) = [0.9, 0.1, 0.0]

Context Vector v(t) — representing the contextual reference field V(t) ("Employee Expectation"):

v(t) = [0.8, 0.2, 0.1]

**Step 2: Calculate Resonance R(t) via Cosine Similarity**

```
  dot product:  m · v = (0.9)(0.8) + (0.1)(0.2) + (0.0)(0.1)
              = 0.720 + 0.020 + 0.000 = 0.740

  ||m(t)|| = sqrt(0.81 + 0.01 + 0.00) = sqrt(0.82) ≈ 0.906
  ||v(t)|| = sqrt(0.64 + 0.04 + 0.01) = sqrt(0.69) ≈ 0.831

  R(t) = (m · v) / (||m|| × ||v||)
       = 0.740 / (0.906 × 0.831)
       = 0.740 / 0.753
       ≈ 0.98   [rounded to 2 decimal places]
```

**Step 3: Calculate Salience S(t) = −ln(p)**

Assume p = 0.10 (probability of this event given discourse history):

S(t) = −ln(0.10) ≈ 2.30

**Step 4: Negentropy Rate Ṅ(t)**

Given value for this illustrative calculation (illustratively assigned):

Ṅ(t) = 0.50

**Step 5: Calculate Coherence Index K(t)**

Using the operational three-term form (Section 3.3) with exploratory weights:

```
  w_R = 1.0   (resonance weight)
  w_N = 0.8   (negentropy weight)
  w_S = 0.2   (salience penalty)

  K(t) = w_R × R(t) + w_N × Ṅ(t) − w_S × S(t)
       = (1.0)(0.98) + (0.8)(0.50) − (0.2)(2.30)
       = 0.98 + 0.40 − 0.46
       = 0.92
```

**Step 6: Diagnosis**

K(t) = 0.92 > 0 ⇒ Diagnosis: STABLE

The discourse window is stable: resonance with the contextual field is high (R = 0.98), the negentropy rate is positive, and the salience penalty does not offset the positive terms. No Cognitive Freeze event is triggered.

━━━━━━━━━━━━━━━━━━━━━━━━━━━━━━━━━━━━━━━━━━━━━━━━━━━━━━━━━━━━━━━━━━━━━━━━

**Sensitivity Remark**

The positive diagnosis is robust to variation in the exploratory weights within a reasonable range. If the salience weight is raised from w_S = 0.2 to w_S = 0.5 while holding the other weights constant, K(t) falls to 0.98 + 0.40 − 1.15 = 0.23 — still positive. The diagnosis would flip to decoherent only if p fell substantially (raising S(t)) or if R(t) fell sharply. These sensitivity conditions are the subject of the empirical calibration procedure specified in Section 5.7 of the main paper.

Note: All values in this walkthrough are illustratively assigned or calculated from stated assumptions. They do not constitute empirical measurements of any real discourse.

━━━━━━━━━━━━━━━━━━━━━━━━━━━━━━━━━━━━━━━━━━━━━━━━━━━━━━━━━━━━━━━━━━━━━━━━

*Mund (2026) — Supplementary Material S2 — Computational Walkthrough*
